# Chitooligosaccharides suppress airway inflammation, fibrosis, and mucus hypersecretion in a house dust mite-induced allergy model

**DOI:** 10.3389/falgy.2025.1533928

**Published:** 2025-01-23

**Authors:** Yun-Ho Kim, Chan-Ho Park, Ju Myung Kim, Yeo Cho Yoon

**Affiliations:** Healthcare & Nutrition Laboratory, Amicogen, Inc., Seongnam, Republic of Korea

**Keywords:** chitooligosaccharides, house dust mites, mucus hypersecretion, airway inflammation, NF-κB pathway

## Abstract

**Background:**

Respiratory allergy is a serious respiratory disorder characterized by inflammation, mucus hypersecretion, and airway tissue sclerosis. Disruption of the T helper 1 (Th1) and T helper 2 (Th2) immune systems by stimuli induced by house dust mites (HDM) and fine particulate matter leads to the secretion of various inflammatory cytokines, resulting in immune respiratory diseases characterized by airway inflammation. Chitooligosaccharides (COS) are known for their antioxidant and anti-inflammatory properties.

**Methods:**

Human airway epithelial cells (BEAS-2B) were cultured in DMEM/F12 medium containing COS at concentrations of 25–100 µg/ml for 24 h. No intracellular toxicity was observed up to 1,000 µg/ml. Cell experiments were conducted at COS concentrations below 100 µg/ml, while animal experiments were performed at concentrations below 100 mg/kg body weight for 4 weeks. Samples of right lung tissue obtained from the experimental animals were used for gene and protein expression analysis, whereas samples of contralateral lung tissue were used for immunohistochemical analysis.

**Results:**

COS regulated Th1 immunity by inhibiting major cytokines, including inflammatory tumor necrosis factor-α (TNF-α), interleukin-1β (IL-1β), and interleukin-6 (IL-6), in BEAS-2B cells. In the HDM-induced allergic respiratory model, COS suppressed the infiltration of inflammatory cells around the airways and inhibited the mRNA expression of Th1 immune cytokines in lung tissues, while also reducing the expression of nuclear factor kappa B (NF-κB)-related proteins. Furthermore, the results confirmed the suppression of the levels of immunoglobulin E (IgE) in the blood secreted by mast cells activated by HDM, which led to a reduction in allergic mucus hypersecretion and airway sclerosis.

**Conclusion:**

In summary, COS are thought to improve airway resistance by alleviating inflammatory allergic respiratory diseases caused by HDM and are regarded as substances that regulate the balance of the Th1 and Th2 immune systems in epithelial cells affected by mucus hypersecretion.

## 1 Introduction

Respiratory allergic diseases, particularly allergic rhinitis and asthma, are a growing global concern (1, 2). These conditions arise from a combination of environmental factors, genetic predisposition, and imbalances in the immune system, with common allergens such as house dust mites (HDM) being recognized as major triggers of airway inflammation and hypersensitivity (3, 4). Such hypersensitivity occurs when the airways overreact to external stimuli, leading to inflammation, bronchoconstriction, and excessive mucus production, which significantly impair respiratory function and the quality of life for affected individuals (5–7).

T cells, which are a crucial component of the immune system, play a central role in the development and progression of respiratory allergic diseases. Additionally, they can contribute to the onset of sclerosis and are implicated in autoimmune diseases such as rheumatoid arthritis, atopy, ulcerative colitis, and fibrosis (8–12). T cells can be divided into two main categories: Th1 and Th2 cells, each driving distinct immune responses. Th1 cells are responsible for cell-mediated immunity, whereas Th2 cells primarily contribute to allergic reactions and responses to parasitic infections. Activation of Th1 cells can provoke inflammatory responses that potentially damage airway epithelial cells. Such damage compromises the airway barrier, facilitating allergen infiltration and exacerbating the release of inflammatory mediators, which in turn worsens airway inflammation (13–15). Th1 cytokines promote the infiltration of inflammatory cells and can induce apoptosis in airway epithelial cells, leading to structural changes in the airways and increasing the risk of developing chronic conditions such as asthma. While excessive activation of Th2 cells plays a significant role in diseases characterized by airway hypersensitivity, the inflammatory mechanisms involving Th1 cells should not be overlooked (16, 17). Th2 cytokines lead to the activation of eosinophils and increased mucus production, aggravating airway inflammation; however, the damage caused by Th1 cell-mediated inflammation is a critical factor in the pathogenesis of these diseases. Therefore, the imbalance between Th1 and Th2 responses plays an essential role in the development of such conditions (6, 18, 19).

Recent studies have highlighted the efficacy of natural compounds in suppressing these inflammatory responses. Among them, chitooligosaccharides (COS) have gained attention owing to their anti-inflammatory and immunomodulatory properties. These chitin-derived compounds exhibit various biological activities, particularly antioxidant, immune-enhancing, and anti-inflammatory effects. Due to these benefits, they are being explored as potential therapeutic agents for respiratory and other inflammatory diseases (20, 21).

This study was designed to investigate the inhibitory effects of COS on airway inflammation induced by allergic hypersensitivity using a mouse model subjected to HDM exposure. Specifically, the aim was to elucidate the mechanisms by which COS suppress airway inflammatory responses and evaluate their therapeutic potential for managing respiratory allergic diseases. This research will provide crucial foundational data regarding the utility of COS in the management of airway inflammatory disorders.

## 2 Materials and methods

### 2.1 Chemicals

DMEM/F12 was provided by the Amicogen Inc. (Jinju, Republic of Korea). All the other reagents were purchased unless otherwise specified. Fetal bovine serum (FBS), penicillin-streptomycin, and trypsin-EDTA were obtained from Lonza (Walkersville, MD, USA). Inhibitor of nuclear factor kappa-B-α (IκB-α) and β-actin antibodies were purchased from Santa Cruz Biotechnology (Dallas, TX, USA), while phosphorylated NF-κB p65 and NF-κB antibodies were obtained from Cell Signaling (Beverly, MA, USA). Essential fatty acid-free bovine serum albumin and skim milk were sourced from Becton Dickinson Company (Sparks, MD, USA). The low-molecular-weight COS used in the experiments were produced directly by Amicogen Inc. (Jinju, Republic of Korea).

### 2.2 BEAS-2B cell culture & viability

Human bronchial epithelial cells (BEAS-2B) were obtained from the American Type Culture Collection (CRL-3588, ATCC) and maintained in DMEM/F12 medium containing 10% FBS and 1% penicillin-streptomycin in a CO_2_ incubator. The MTT assay was performed to assess cytotoxic effects. The cells were seeded at a density of 1 × 10^4^ cells/well in a 96-well plate and treated with HDM or COS. After 24 h of incubation, 5 mg/ml of MTT reagent was added, and the cells were incubated for an additional 2 h. Subsequently, the culture was centrifuged at 2,000 rpm for 10 min at 4°C, and the supernatant was discarded. DMSO was then added to each well, and the absorbance was measured at 540 nm using a microplate reader (Thermo Scientific, Waltham, MA, USA).

### 2.3 Animal experiments

This study was approved by the Institutional Animal Care and Use Committee of Jeonbuk National University Hospital (JBUH-IACUC-2022-28) and was conducted in compliance with the University's guidelines for the care and use of experimental animals. The following conditions were maintained in the experimental environment: temperature of 23°C ± 3°C and relative humidity of 55% ± 15%.The lighting cycle lasted 12 h, at an intensity between 150 and 300 Lux. These environmental parameters were regularly monitored throughout the breeding period. The animals were provided with food obtained from the Central Experimental Animal Center (Seoul, Republic of Korea) and had free access to it. Filtered water was supplied in polycarbonate bottles, and was also freely available. Bedding materials were sourced from Coretech Co., Ltd. (Pyeongtaek, Republic of Korea). During the adaptation, treatment, and blood collection phases, the rodents were housed in polycarbonate cages (235 mm wide × 380 mm long × 175 mm high), with a maximum of five animals per cage. Cages, bedding, and water bottles were changed at least once a week. Prior to infection, the animals were weighed, ranked, and randomly assigned to groups to ensure uniform average body weight within each group.

To establish an HDM-induced airway allergic inflammation model, freeze-dried HDM extract (14-4107-01, Thermo fisher) was reconstituted in saline at a concentration of 2 μg/μl. Following acclimation, the animals were anesthetized, and the extract was administered four times at 1-week intervals at a dose of 100 μg/50 μl per mouse using a small animal ventilator (SAR-1000, CWE, Inc.) maintaining a respiratory rate of 80 bpm and a tidal volume of 0.18 ml. The experiment was conducted for five groups, each consisting of eight mice and defined as follows: control, HDM, HDM + 10 mg/kg/body weight (BW), HDM + 20 mg/kg/BW, and HDM + 100 mg/kg/BW. For the control group, which did not receive any disease-inducing treatment, an equivalent volume of saline solution (50 μl) was administered using the same equipment.

After administration, the trachea of each mouse was intubated, and the lungs and airways were rinsed with 1 ml of PBS, before collecting a sample of bronchoalveolar lavage fluid (BALF). The number of inflammatory cells was determined using a Hemavet HV950 multispecies hematologic analyzer (Drew Scientific, Oxford, CT, USA). The right lung was collected, frozen in liquid nitrogen, and stored at −80℃ until use for real time (RT)-PCR and Western blotting. The left lung was fixed in 4% paraformaldehyde and used for immunohistochemical analysis.

### 2.4 Cytokine RNA expression analysis

Cell and lung tissue samples were processed to synthesize cDNA from 5 μg of total RNA using the High-Capacity cDNA Reverse Transcription Kit (Thermo Fisher Scientific). The reaction mixture for RT-PCR contained 1 μl of the synthesized cDNA, 1 μl of TaqMan probes, 10 μl of TaqMan Universal Master Mix II (Thermo Fisher Scientific), and 8 μl of nuclease-free water. The TaqMan genes used for quantitative PCR (qPCR) were obtained from Applied Biosystems. The RT-PCR conditions included an initial step at 50°C for 2 min, followed by one cycle at 95°C for 10 min, and then 40 cycles of denaturation at 95°C for 15 s and annealing at 60°C for 15 s. PCR primers were used based on the information provided in Table 1.

**Table 1:** PCR primer sequence.

Gene name	Primer sequences (5’-3’)
TNF-α	Forward 5’-CAGGAGAAAGTCAGCCTCCTC-3’
	Reverse 5’-CCAGGTACATGGGCTCATACC-3’
IL-1β	Forward 5’-TGGCAGCTACCTATGTCTTGC-3’
	Reverse 5’- CAGTGCAGCTGTCTAATGGGA-3’
IL-6	Forward 5’- AAGAGACTTCCAGCCAGTTGC-3’
	Reverse 5’- TGGTCTGTTGTGGGTGGTATC-3’
GAPDH	Forward 5’- CATGGCCTTCCGTGTTC-3’
	Reverse 5’- CTGCTTCACCACCTTCTT-3’

TNF, tumor necrosis factor; IL, Interleukin; GAPDH, glyceraldehyde 3-phosphate dehydrogenase.

### 2.5 Western blot analysis

Cell and lung tissue samples were washed twice with PBS, and proteins were extracted using NE-PER nuclear and cytoplasmic extraction reagents (Cat#: 78835, Thermo Fisher). The extracted proteins were quantified using Bradford reagent (Bio-Rad, Hercules, CA, USA). Then, they were mixed with sample buffer and heated at 95°C for 5 min, followed by separation using 10%–12% SDS-PAGE. After separation, the proteins were transferred to a PVDF membrane using a semi-dry transfer system at 15 V for 60 min. The membrane was then incubated with blocking buffer (5% skim milk in 1 × TBST) for over 1 h, followed by incubation with the primary antibody at 4°C overnight. The membrane was washed five times with 1 × TBST at 7-min intervals. Subsequently, it was incubated with the secondary antibody at room temperature for over 1 h, followed by another five washes with 1 × TBST at 7-min intervals. Finally, the membrane was developed using an enhanced chemiluminescent (ECL) reagent and exposed to an x-ray film.

### 2.6 Biochemical analysis of blood samples

Mice that had fasted for more than 14 h, all the mice, were anesthetized with ketamine, and 1 ml of blood was collected via cardiac puncture. The collected blood was centrifuged at 3,000 rpm for 10 min to obtain serum, which was transferred to EP tubes and stored at −80°C. Biochemical analyses for triglycerides, total cholesterol, aspartate aminotransferase (AST), and alanine aminotransferase (ALT) were performed. The reagents for these analyses, i.e., triglyceride measurement reagent (AM-157S-K), cholesterol measurement reagent (AM 202-K), GOT reagent (AM-103 K), and GPT reagent (AM-102 K), were purchased from Asan Pharmaceutical (Seoul, Republic of Korea). The levels of IgE in the collected plasma were measured using the mouse IgE ELISA kit (ab157718, Abcam).

### 2.7 Measurement of airway resistance

Mice were anesthetized with a mix of ketamine (125 mg/kg) and medetomidine 0.4 mg/kg) and ventilated with O_2_/air (1:2) at a rate of 150 beats/min (TV = 0.3 ml). Pressure was measured by placing a small catheter in the trachea of each mouse. Methacholine (acetyl-β-methyl-choline chloride; Sigma) was administered in increasing doses (0–50 mg/ml, delivered as 10% puffs over 10 s) via an aerosol generated by a nebulizer (EMKA Technologies, Paris, France). The nebulizer was connected to the animal the FlexiVent device (Flexivent FV-FX2, SCIREQ). After the initial dose of methacholine, lung resistance was measured for 3 min, and this procedure was repeated for each subsequent dose (22).

### 2.8 Histological staining of lung tissues

Small lung tissue samples were collected at the end of the experiments and fixed in 10% formalin for histological analyses. Once embedded in paraffin, these samples were cut into sections with a thickness of 5 μm and deparaffinized. Depending on the specific staining required, the sections were subjected to one of the following staining procedures:

(1) Harris hematoxylin and eosin Y (H&E) staining: The sections were first stained with Harris hematoxylin for 2 min and then with eosin Y.
(2) Periodic Acid-Schiff (PAS) staining: The sections were deparaffinized and stained with alcian blue (Sigma–Aldrich) for 15 min, followed by thorough washing under running tap water for 2 min.
(3) Sirius Red staining: The sections were immersed in Sirius Red solution for 1 h to stain collagen, followed by rinsing in acetic acid solution and water for approximately 5 min.
(4) Masson's trichrome staining: The sections were stained using this technique to observe the degree of fibrosis in the subepithelial tissue.

After the applied staining procedures, the sections were dehydrated through a series of 50%, 70%, 90%, 95%, and 100% ethanol and xylene. After adding a drop of mounting solution and placing a cover slide on the top. The stained tissue slides were observed under an optical microscope BX41 (Olympus, Tokyo, Japan).

### 2.9 Statistical analysis

In the statistical analyses, the interquartile range method was employed to exclude outliers. The interquartile range is defined as the difference between the first quartile (Q1) and the third quartile (Q3). Values that exceeded the established criteria for outliers were excluded from the analysis. Results are presented as the mean ± SE for each treatment group across all experiments. Statistical analyses were performed in SPSS v. 18.0 (IBM Corp., Armonk, NY, USA). All data measurements were tested for normality using the Kolmogorov-Smirnov and Shapiro-Wilk methods, followed by comparisons between groups. Based on the normality results, significance was assessed using the ANOVA method, with *post hoc* testing performed via the *t*-test. In cases where normality was not met, significance was evaluated using the Kruskal-Wallis test, with *post hoc* testing conducted using the Mann-Whitney *U*-test. Significance levels for the *t*-test were defined as ^#^*p* < 0.05, ^##^*p* < 0.01, and ^###^*p* < 0.001, while significance for the U test was defined as *p < 0.05, **p < 0.01, and ***p < 0.001.

## 3 Results

### 3.1 Assessment of COS safety based on cellular and blood parameters

**Figure 1:**
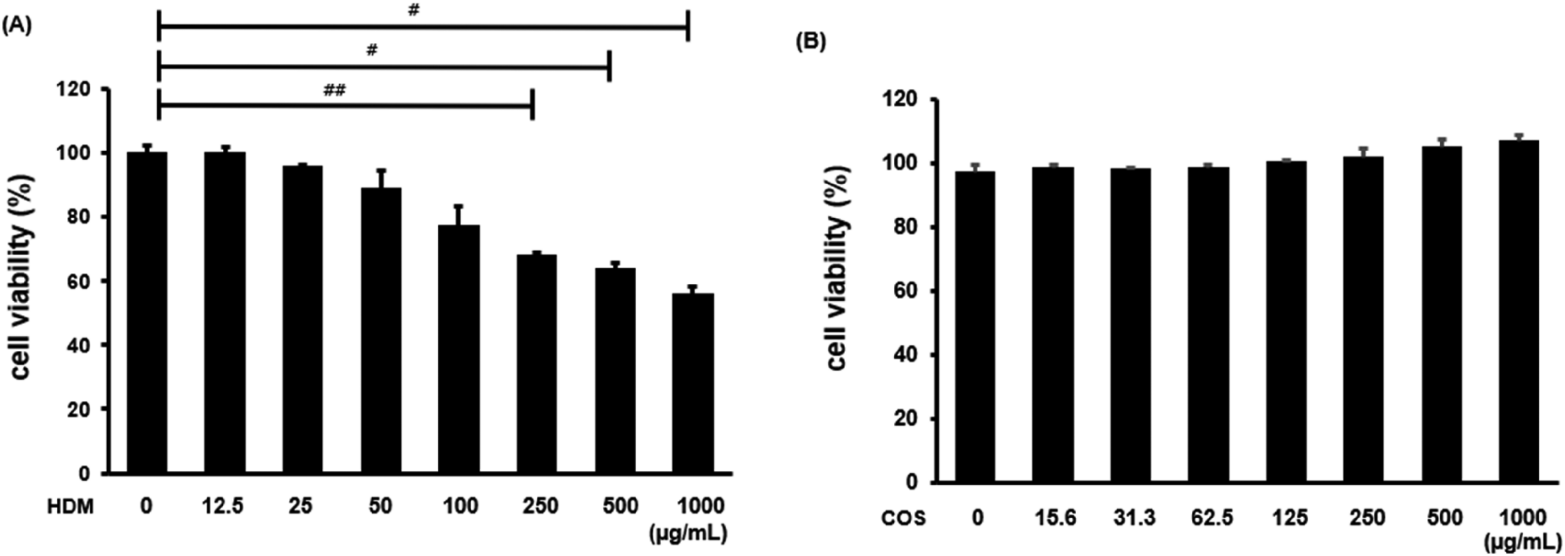
Cytotoxicity of HDM and COS. BEAS-2B cells were treated with HDM or COS at concentrations ranging from 12.5 to 1,000 µg/ml, and cytotoxicity was assessed using the MTT assay **(A,B)**. In each bar graph, values (mean ± SEM, *n* = 3) were compared among the HDM groups. The significance level was determined via *t*-test. Data are expressed as the mean ± SE, with significance levels set at ^#^*p* < 0.05, ^##^*p* < 0.01, and ^###^*p* < 0.001.

The toxicity assessment was conducted on airway epithelial cells treated with HDM and COS. Specifically, BEAS-2B cells were treated with HDM at concentrations ranging from 12.5 to 1,000 μg/ml for 24 h, and cell viability was then evaluated using the MTT assay. As shown in Figure 1A, cell viability was 77% after treatment with 100 μg/ml HDM. Since HDM are known to simultaneously induce cell death and inflammation, their concentration was set at 100 μg/ml in subsequent experiments (23, 24). No toxicity was observed at COS concentrations below 1,000 μg/ml (Figure 1B). To evaluate the safety of orally administering COS to animals, body weight was measured at the end of the 4-week experiment. No significant differences in body weight were observed between groups, and while overall lung weight increased in the HDM-exposed group, no significant changes were noted after COS ingestion. Among the measured hematological indexes, which included aspartate aminotransferase (AST), alanine aminotransferase (ALT), triglycerides (TG), and total cholesterol (TC), AST decreased in animals treated with high doses of COS, and the same tendency was observed for TG and TC in this group. These findings confirmed that COS have a slight impact on hematological indexes but remain safe (Table 2).

**Table 2:** Measurement of changes in body weight and blood parameters.

Measurement parameters	Control	HDM	HDM + COS 10	HDM + COS 20	HDM + COS 100
Initial body weight (g)	18.02 ± 0.38	17.92 ± 0.37	17.98 ± 0.32	17.94 ± 0.33	17.78 ± 0.22
Final body weight (g)	19.50 ± 0.47	20.02 ± 0.30	19.79 ± 0.24	19.20 ± 0.27	19.36 ± 0.18
ALT (U/L)	77.25 ± 9.29	84.30 ± 10.12	73.00 ± 4.46	71.30 ± 4.29	57.58 ± 10.45
AST (U/L)	93.86 ± 13.55	104.63 ± 5.45	97.25 ± 6.90	107.13 ± 5.57	81.71 ± 6.35^#^
TG (mg/dl)	81.98 ± 10.28	99.05 ± 25.67	65.37 ± 10.36	84.20 ± 6.77	49.60 ± 15.64
TC (mg/dl)	98.71 ± 9.13	124.95 ± 12.97	133.80 ± 12.84	107.75 ± 7.90	108.58 ± 12.83

The significance level was determined using ANOVA, followed by Duncan's *post hoc* test. Values (mean ± SEM, *n* = 7–8) that do not share lowercase letters indicate significant differences at *p* < 0.05. Additionally, *t*-tests were conducted to compare the HDM-treated groups. Data are presented as the mean ± SE, with significance levels set at ^#^*p* < 0.05, ^##^*p* < 0.01, and ^###^*p* < 0.001.

AST, aspartate aminotransferase; ALT, alanine aminotransferase; TG, triglyceride; TC, total cholesterol.

### 3.2 Inhibition of airway hyperresponsiveness by COS

**Figure 2:**
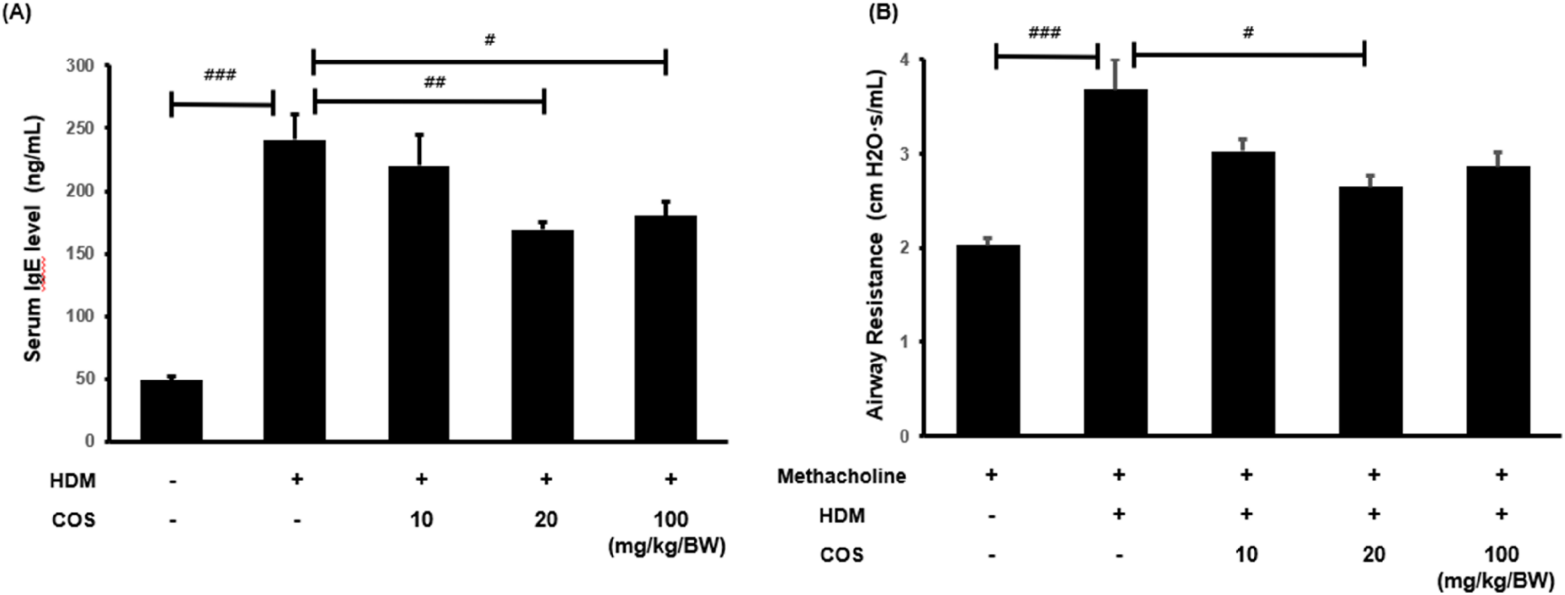
COS intake inhibited airway obstruction and hyperresponsiveness in a model of HDM-induced bronchial damage. In the HDM-treated animals, the concentration of IgE promoting histamine release in the serum was determined to confirm airway obstruction and hyperresponsiveness after COS administration at 10–100 mg/kg/BW **(A)** Methacholine, which causes airway constriction, was injected at a concentration of 25 mg/ml, and airway resistance for the respective respiration was measured by maintaining breathing by connecting the animal to the FlexiVent device **(B)** The significance level was determined via ANOVA test, followed by *t*-test *post hoc* test. In each bar graph, values that do not share lowercase letters indicate significant differences at *p* < 0.05. Additionally, *t*-tests were employed to compare the HDM-treated groups. Data are expressed as the mean ± SE, with significance levels set at ^#^*p* < 0.05, ^##^*p* < 0.01, and ^###^*p* < 0.001 [mean ± SEM, *n* = 5**(A)**, *n* = 8 **(B)**].

The IgE levels in mouse plasma samples obtained via blood centrifugation were analyzed using the mouse IgE ELISA kit. The HDM-treated mice exhibited significantly higher IgE levels than the control group. In contrast, a significant decrease in this parameter was observed in the group administered with COS at 20 mg/kg/BW compared with the HDM-treated group (Figure 2A). Elevated IgE levels are known to cause mucus hypersecretion and increased airway resistance (25). The latter reaction was specifically assessed by administering methacholine, which induces airway constriction. The results showed that airway resistance increased in the HDM-treated mice, while it significantly decreased in the group treated with COS concentrations > 20 mg/kg/BW (Figure 2B).

### 3.3 Suppression of airway mucus hypersecretion and fibrosis by COS

**Figure 3:**
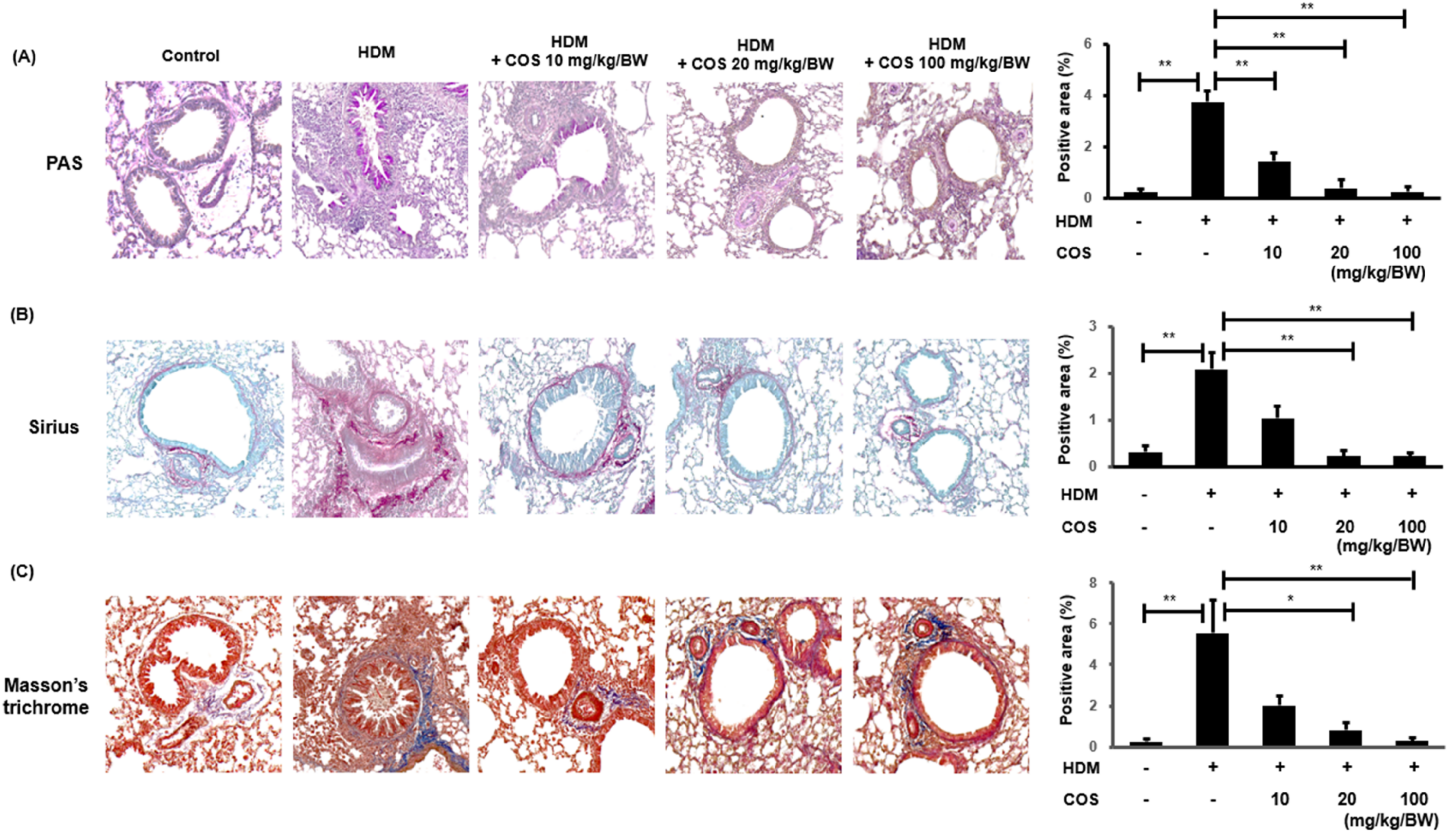
COS intake inhibited mucus hypersecretion and airway fibrosis induced by HDM treatment. Airway mucus hypersecretion was confirmed via PAS staining, which highlights acidic mucus in a reddish-purple hue. Airway fibrosis was assessed using Sirius Red and Masson's trichrome staining after COS administration at doses ranging from 10 to 100 mg/kg/BW **(A)** In the HDM-treated animals, airway fibrosis was evaluated using the same staining methods applied after COS treatment. Sirius Red and Masson's trichrome staining techniques highlight fibrotic collagen; the former in red, the latter in blue **(B,C)**. The images depict the stained lung tissues of five mice, and the bar graphs (created in ImageJ) represent the quantified stained area as a percentage of the total area. The significance level was determined via Kruskal-Wallis test, followed by Mann-Whitney *U*-test *post hoc* test. In each bar graph, values that do not share lowercase letters indicate significant differences at *p* < 0.05. Additionally, Mann-Whitney *U*-tests were employed to compare the HDM-treated groups. Data are expressed as the mean ± SE, with significance levels set at *p < 0.05, **p < 0.01, and ***p < 0.001.

The effect of COS in terms of suppressing the excessive secretion of airway mucus was evaluated through PAS staining of lung tissue sections. Mucus secretion increased with HDM exposure; however, microscopic examination showed that mucus accumulation decreased in the groups that received COS at doses > 20 mg/kg/BW. Airway sclerosis is characterized by the thickening of airway tissues due to inflammation and fibrosis, with collagen fiber accumulation playing a crucial role. Sirius Red or Masson's trichrome staining can be employed to observe increases in collagen fibers or their abnormal arrangement, which are associated with the thickening of the airway wall. The infiltration of inflammatory cells and the increase in collagen fibers occur simultaneously, reflecting the pathological features of airway sclerosis (Figure 3A) (26, 27). In the HDM-treated group, fibrosis was noted around the smooth muscle of the airway, while in the group administered with COS, airway sclerosis was suppressed (Figure 3B,C).

### 3.4 Inhibition of inflammatory cell accumulation by COS

**Figure 4:**
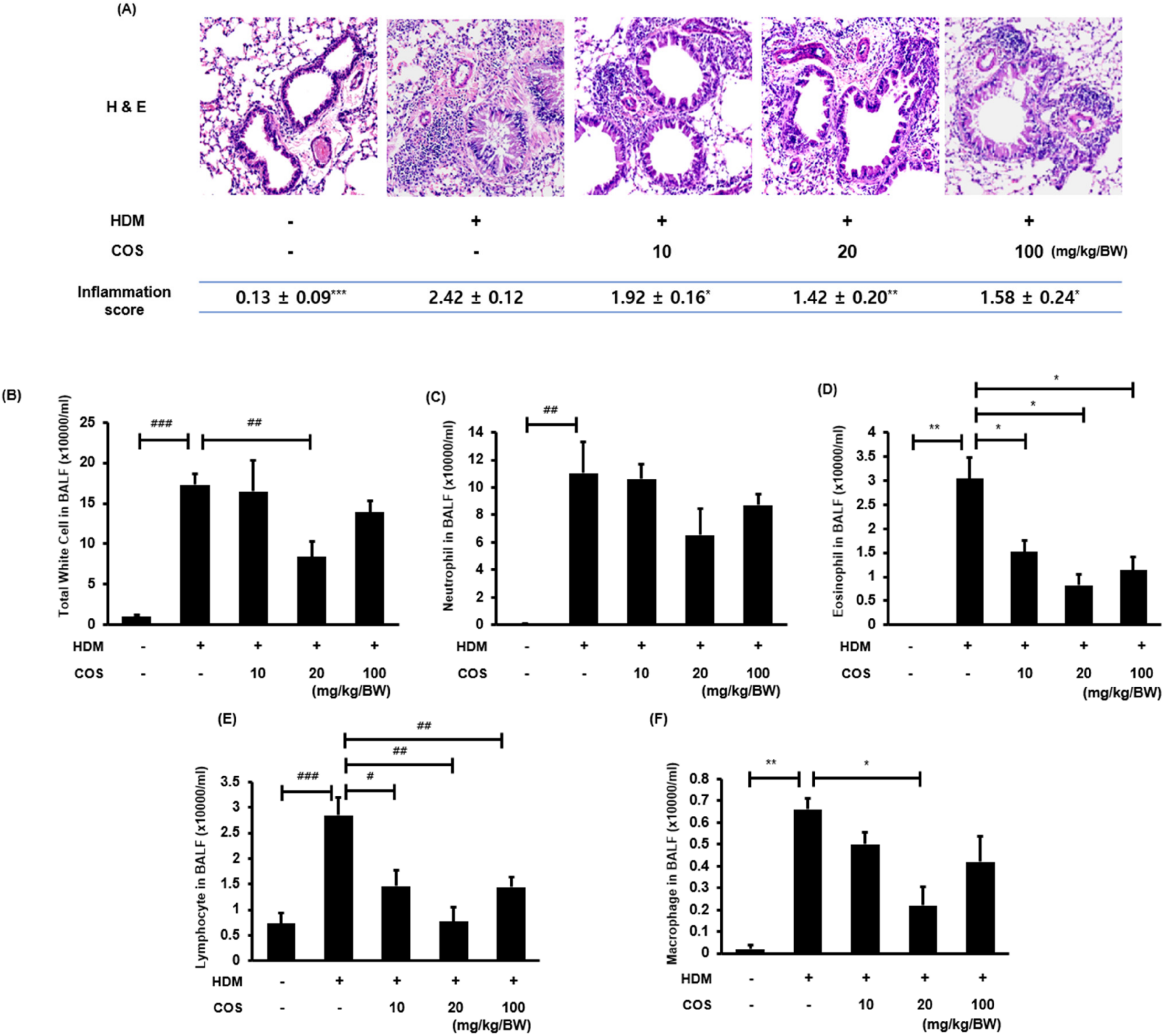
Anti-inflammatory effect of COS on the lung tissues of mice exposed to HDM. Representative images of H&E-stained lungs were visually assessed by three independent investigators, who assigned scores for the levels of inflammation using a 0–3 scale: 0, normal; 1, mild; 2, severe, and 3, very severe (the mean value is indicated accordingly in the figure) **(A)** After euthanasia, BALF was obtained via aspiration through the trachea, and immune cells, including leukocytes and other immune cells, were counted using an HV950 Multispecies Hematologic Analyzer **(B**–**F)**. The significance level was determined via ANOVA or Kruskal-Wallis test, followed by *t*-test or Mann-Whitney *U*-test *post hoc* test [mean ± SEM, *n* = 4**(A)**, *n* = 7 **(B**–**F)**]. Additionally, *t*-tests or Mann-Whitney *U*-tests were employed to compare the HDM-treated groups. Data are expressed as the mean ± SE, with significance levels indicated as follows: [*t*-test: ^#^*p* < 0.05, ^##^*p* < 0.01, and ^###^*p* < 0.001] [Mann-Whitney *U*-test: *p < 0.05, **p < 0.01, and ***p < 0.001].

H&E staining revealed the differences in the infiltration of inflammatory cells in lung tissues between the control and HDM-treated mice. Significant pathological features of inflammatory cell infiltration were observed around the small airways in the mice exposed to HDM. The evaluation was conducted by three experts, who scored the level of inflammation on a 0–3 scale. Better scores were assigned to the group treated with COS at > 20 mg/kg/BW (Figure 4A). The number of inflammatory cells measured in the BALF, including eosinophils, neutrophils, lymphocytes, and macrophages, decreased under COS administration at doses greater than 10 mg/kg/BW (Figures 4B–F). This suggested that the reduction in the number of inflammatory cells was associated with decreased inflammatory cell infiltration.

### 3.5 Inhibition of inflammatory cytokines and the NF-κB pathway by COS

**Figure 5:**
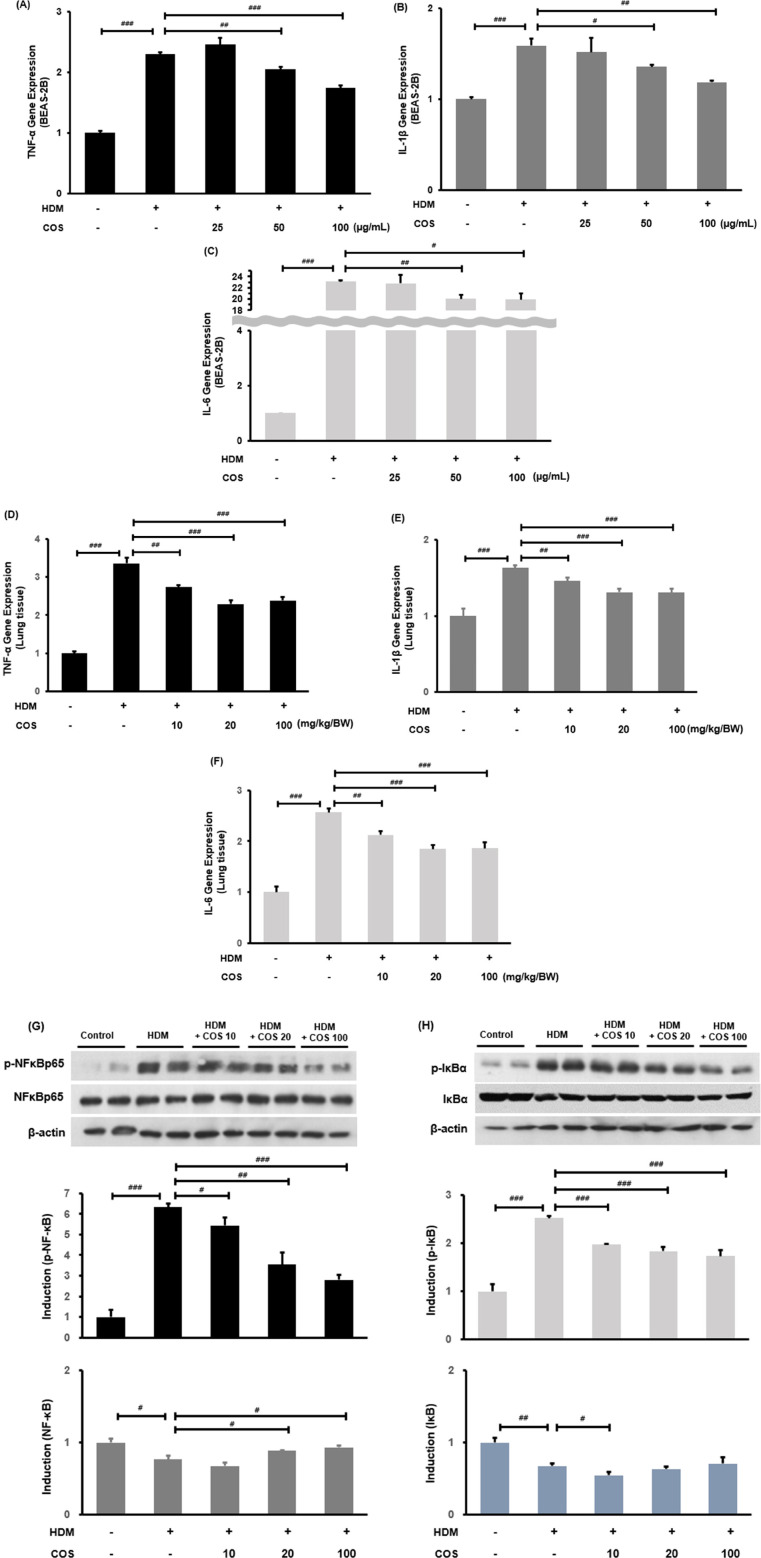
COS intake inhibited inflammatory cytokines and the NF-κB pathway. RT-PCR confirmed that COS intake inhibited TNF-α, IL-1β, and IL-6 mRNA expression in BEAS-2B cells exposed to HDM **(A**–**C)**. Additionally, COS at concentrations greater than 10 mg/kg/BW was shown to have the same inhibitory effect in the lung tissues of HDM-treated animals **(D**–**F)**. The NF-κB pathway was assessed through protein expression analysis using Western blotting in the same lung tissues **(G,H)**. β-Actin protein was used as an internal control. The significance level for each bar graph was determined via ANOVA, followed by *t*-test *post hoc* test. Additionally, *t*-tests were employed to compare the HDM-treated groups. Data are expressed as the mean ± SE, with significance levels set at ^#^*p* < 0.05, ^##^*p* < 0.01, and ^###^*p* < 0.001 [mean ± SEM, *n* = 4–5].

In the airway epithelial cells treated with HDM, the RNA expressions of TNF-α, IL-1β, and IL-6 increased, while they began to decrease at COS concentrations > 50 µg/ml (Figures 5A–C). The mRNA expression of inflammatory cytokines induced by HDM was significantly suppressed in the lung tissues of experimental mice treated with COS (Figures 5D–F). It was also found that HDM promoted the phosphorylation of IκB, leading to increased nuclear translocation of NF-κB, which regulates a key mechanism related to inflammation. The COS treatment inhibited the phosphorylation of IκB and NF-κB, thereby counteracting the inflammatory signaling pathway of NF-κB and ultimately reducing inflammation in the airways (Figures 5G,H).

## 4 Discussion

**Figure 6:**
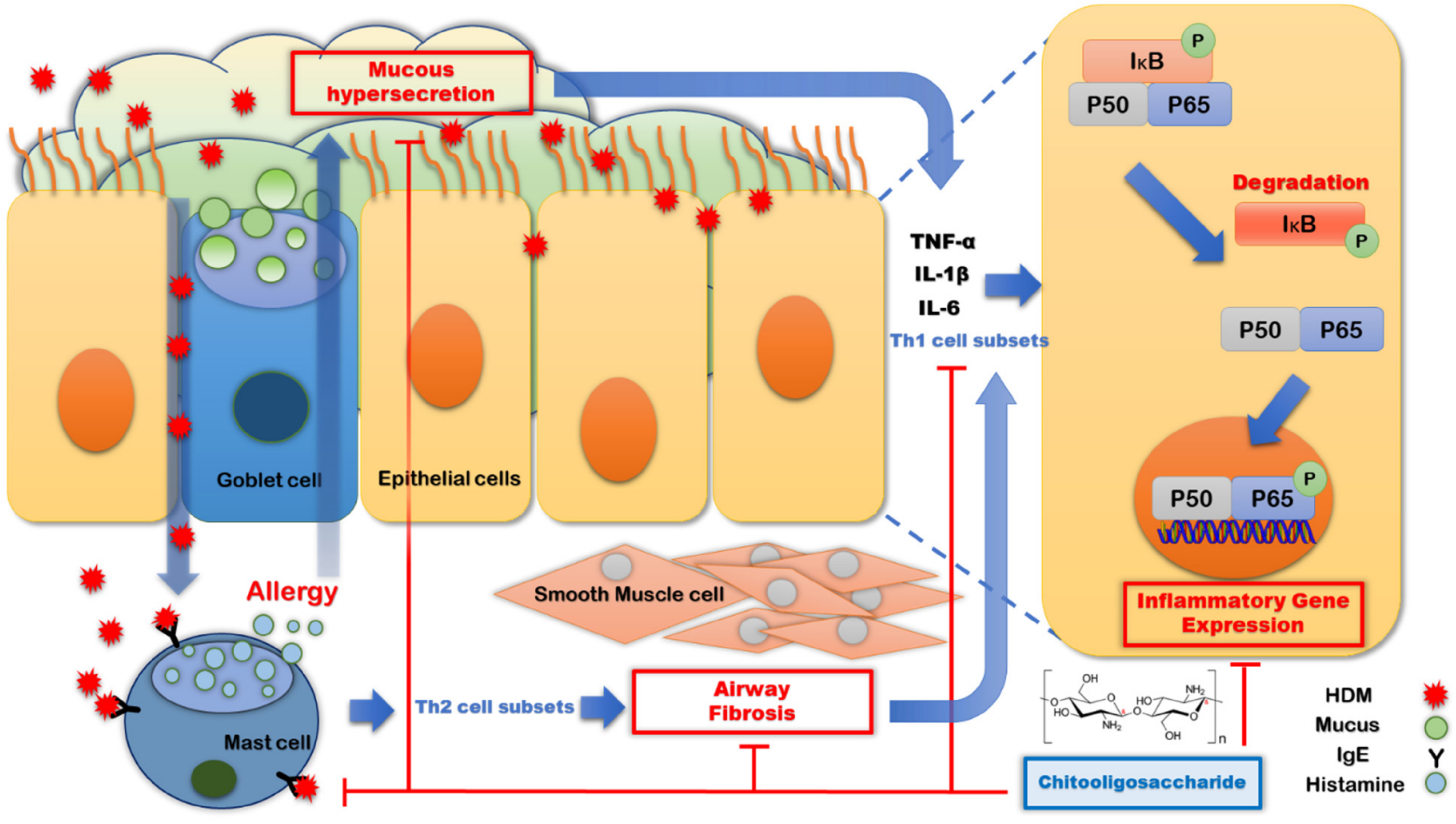
Mechanism through which COS suppress allergic immune respiratory diseases. The introduction of HDM into the respiratory tract stimulates IgE secretion and causes allergies by inducing histamine release from mast cells, leading to airway sclerosis and mucus hypersecretion. This results in allergic inflammation in airway epithelial cells. COS intake suppresses mucus hypersecretion and airway sclerosis by inhibiting IgE secretion, ultimately reducing the expression of Th1-related inflammation in airway epithelial cells and demonstrating its effect in suppressing allergic airway inflammation.

The present study revealed four key mechanisms through which COS effectively improved allergic respiratory inflammation. Specifically, COS (1) inhibited the increase in IgE levels induced by HDM exposure; (2) suppressed excessive mucus secretion caused by allergies and prevented airway fibrosis, thereby improving airway resistance; (3) inhibited the infiltration of inflammatory cells into the airways, and (4) suppressed the expression of Th1-type inflammatory cytokines and the activation of the NF-κB pathway in airway epithelial cells and lung tissue (Figure 6).

HDM exposure leads to intense stimulation of airway epithelial cells via the FGFR/MAPK/NF-kB and STAT3/NF-kB pathways, resulting in a robust Th1 immune response. This leads to increased expression of the IL-8 and AP-1 genes, which are critical mediators of the inflammatory response (28–30). Persistent inflammation significantly raises the likelihood of developing allergic asthma, resulting in a decline in respiratory function and symptoms such as coughing and shortness of breath (31, 32). The respiratory system responds to external substances by producing pro-inflammatory cytokines such as TNF-α and IFN-γ through CD4 and CD28 interactions, stimulating T cells. Concurrently, HDM stimulate B cells to secrete IgE, which binds to receptors on mast cells, resulting in the release of allergy-inducing substances like histamine. Recent studies have shown that mast cells produce significant amounts of cytokines such as IL-6, further stimulating B cells and enhancing IgE production. If this immune response persists, it can lead to chronic inflammation, exacerbating respiratory diseases such as asthma (33, 34).

An interesting finding from this experiment was that COS exhibited a concentration-dependent effect on serum IgE and airway resistance at low doses (≤20 mg/kg/BW), but this effect was reduced or eliminated at higher doses (≤100 mg/kg/BW) (Figure 2A,B). Fong et al. reported that high concentrations of chitosan (150 µg/ml; ≥3 kDa, 98% deacetylation) triggered the activation of the inflammasome in macrophages, resulting in increased release of IL-1β and prostaglandin E2 (PGE2). At the same time, this high concentration inhibited the secretion of interleukin-1 receptor antagonist (IL-1ra) and C-X-C motif chemokine ligand 10 (CXCL10/IP-10). Conversely, lower concentrations of chitosan (5–50 µg/ml) produced the opposite effects (35). High doses of ingested chitooligosaccharides were recognized as foreign invaders, and the Th1 immune response was not diminished. In this experiment, the expression of Th1 immune markers was effectively confirmed at 20 mg/kg/BW, but it did not show a significant effect at a concentration of 100 mg/kg (Figures 5A–F). Therefore, it is considered important to ingest chitooligosaccharides at an appropriate dose.

HDM- and ovalbumin-treated animal models are widely used for studying allergic asthma. Both models have been shown to exhibit a persistent stimulation of eosinophils, leading to the overexpression of mucus-secreting genes like MUC5AC within the airways (26, 36). This results in excessive mucus production and significant infiltration of inflammatory cells into the lungs, contributing to inflammation (30, 37, 38). Mucus hypersecretion can also occur due to SARS-CoV-2 infection (39, 40). In the case of HDM, these allergens activate Th2 immune cells, causing mucus hypersecretion and airway fibrosis (41–43). Although fibrosis activates Th1 cytokines and interleukins to restore immune balance, it can also induce inflammation in epithelial cells. Our experiment confirmed that exposing airway epithelial cells to HDM at 100 µg/ml increased Th1 immune responses, including TNF-α, IL-1β, and IL-6 production. In contrast, these were inhibited at COS concentrations above 50 µg/ml. Th1 immune activity induced by fibrosis may lead to airway damage, but COS are considered to be capable of mitigating such response.

As naturally derived compounds, COS exhibit significant anti-inflammatory effects across various tissues, including the small intestine, neurons, and blood vessels (20, 44–46). In an animal model of inflammatory bowel disease similar to the model used in the present study, COS have been shown to suppress intestinal inflammation by inhibiting IL-6 cytokines and the TLR-4/NF-kB/MAPK pathways while activating Th2 cytokines such as IL-4 and IL-10, thus improving intestinal mucus production (47, 48). Airway mucus, whose composition is similar to that of intestinal mucus, primarily serves to protect against bacteria and external invaders, as well as to induce immune responses against infections (49, 50). Excessive mucus production in the airways can lead to dyspnea and airway fibrosis (6, 51). Persistent inflammatory responses can result in smooth muscle contraction and diminished airway mobility, contributing to breathing difficulties in respiratory diseases (52). COS have been shown to inhibit airway fibrosis, with improvements observed at doses > 20 mg/kg/BW.

However, limited research has been conducted on the mechanisms through which COS affect mucus secretion in the airways. Previous studies have confirmed that COS have virucidal effects against viruses such as COVID-19 and influenza and effectively inhibit the p38 MAPK inflammatory pathway induced by respiratory viral infections (53, 54). This anti-inflammatory effect likely results from the regulation of immune cell activation and suppression of inflammatory mediator secretion by COS. In the present study, histological analysis using PAS staining revealed that mucus hypersecretion was inhibited in mice treated with COS at a concentration of 20 mg/kg/BW. Respiratory mucus suppression indicates an improvement in disease. Mucus secretion is interpreted as a contrasting effect between the airway and small intestine. In the small intestine, where intestinal microbes are abundant, increased mucus secretion signifies an improvement in intestinal health. Indicating contrasting mechanisms in the intestine and airways, which are related to recent microbiome research.

Previous studies have shown that gut microbiota interact with the lungs to regulate the immune system in various ways (55, 56). These microorganisms play a role in asthma as well as in various allergic diseases, particularly affecting allergic sensitization. Epidemiological studies using animal models have indicated that decreased gut bacterial diversity is associated with increased rates of allergic sensitization (57–59). COS, which are produced through enzymatic hydrolysis, stimulate the growth of *Lactobacillus* and *Bifidobacterium* species in a concentration-dependent manner (60). They also maintain the stability of gut microbial structures, reducing the relative abundance of *Bifidobacterium* spp., *Eubacterium rectale, Clostridium histolyticum*, and *Bacteroide*s while increasing the *Lactobacillus*/*Enterococcus* ratio (61). Therefore, COS appear to selectively promote beneficial microorganisms, including lactic acid bacteria and bifidobacteria. However, the mechanisms through which these compounds regulate gut microbiota and their metabolites need to be further investigated.

In this study, we confirmed that the intake of COS improved respiratory diseases. Given that the roles of mucus in the small intestine and airway are different and that ingested COS affects intestinal microflora, we plan to conduct additional experiments to investigate the metabolic aspects of COS action in the small intestine, as well as the route through which it is absorbed and exerts its effects on the respiratory system.

In conclusion, the results of this study indicated that COS inhibit the NF-κB pathway, as well as TNF-α, IL-1β, and IL-6 production, in airway epithelial cells, reduce IgE levels in the blood, suppress mucus hypersecretion, and improve airway fibrosis, potentially alleviating airway hyperresponsiveness and inflammation in allergic asthma.

## Data Availability

The datasets presented in this study can be found in online repositories. The names of the repository/repositories and accession number(s) can be found in the article/Supplementary Material.
